# Soft tissue extension increases the risk of local recurrence after curettage with adjuvants for giant-cell tumor of the long bones: A retrospective study of 93 patients

**DOI:** 10.3109/17453674.2012.711193

**Published:** 2012-08-25

**Authors:** L van der Heijden, MA van de Sande, PD Dijkstra

**Affiliations:** Department of Orthopaedic Surgery, Leiden University Medical Center, Leiden, the Netherlands

## Abstract

**Background and purpose:**

Risk factors for local recurrence of giant-cell tumor of bone (GCTB) have mostly been studied in heterogeneous treatment groups, including resection and intralesional treatment. The aim of the study was the identification of individual risk factors after curettage with adjuvants in GCTB.

**Methods:**

Of 147 patients treated for primary GCTB between 1981 and 2009, 93 patients were included in this retrospective single-center study. All patients were treated with curettage and polymethylmethacrylate (PMMA) with (n = 75) or without (n = 18) phenol. Mean follow-up was 8 (2–24) years. Recurrence-free survival was assessed for treatment modalities. Age, sex, tumor location, soft tissue extension, and pathological fractures were scored for every patient and included in a Cox regression analysis.

**Results:**

The recurrence rate after the first procedure was 25/93. Recurrence-free survival for PMMA and phenol and for PMMA alone was similar. Eventually, local control was achieved using 1 or multiple intralesional procedures in 85 patients. Resection was required in 8 patients. A higher risk of local recurrence was found for soft tissue extension (HR = 5, 95% CI: 2–12), but not for age below 30, sex, location (distal radius vs. other), or pathological fracture.

**Interpretation:**

Curettage with adjuvants is a feasible first-choice treatment option for GCTB, with good oncological outcome and joint preservation. Soft tissue extension strongly increased the risk of local recurrence, whereas age, sex, location, and pathological fractures did not.

Giant-cell tumor of bone (GCTB) is aggressive locally with recurrence rates of 27–65% after curettage with bone grafting (Campanacci et al. 1987, Balke et al. 2008), 12–27% after curettage with adjuvants such as high-speed burr, phenol, and polymethylmethacrylate (PMMA) (Balke et al. 2008, Becker et al. 2008, Kivioja et al. 2008), and 0–12% after en bloc resection (Balke et al. 2008, Errani et al. 2010). In clinical practice, the choice of surgical procedure depends mostly on the feasibility of curettage and cementation vs. resection, but in part also on the expected risk of local recurrence in individual patients. Cortex destruction, soft tissue extension, pathological fractures, young age, and location in the distal radius have been suggested to be risk factors for local recurrence (O’Donnell et al. 1994, Prosser et al. 2005, Becker et al. 2008, Balke et al. 2008, Kivioja et al. 2008, Errani et al. 2010, Klenke et al. 2011), but these have not been confirmed by others (Trieb et al. 2001, Turcotte et al. 2002, Saiz et al. 2004). Most studies that aimed at identification of risk factors for local recurrence have included both resection and curettage in risk analyses (Balke et al. 2008, Becker et al. 2008, Kivioja et al. 2008, Klenke et al. 2011), which results in a selection bias because of lower recurrence rates after resection.

At our tertiary referral center for musculoskeletal oncology, curettage with phenol and PMMA is the preferred standard treatment for all GCTB. Over 75% of patients with primary or recurrent GCTB presenting at our center underwent curettage with adjuvants; the rest underwent en bloc resection. The indications for resection were location in the axial skeleton or severe joint destruction. In contrast to other studies (McDonald et al. 1986, Su et al. 2004, Kivioja et al. 2008, Errani et al. 2010), soft tissue extension and pathological fractures were not contraindications for intralesional treatment; this is in accordance with extended indications for curettage as described previously (Campanacci et al. 1987, Turcotte et al. 2002, Balke et al. 2008, Becker et al. 2008, Klenke et al. 2011). The advantage is avoidance of prosthetic reconstruction at a relative young age. The disadvantage may be an increased risk of local recurrence. We retrospectively evaluated risk factors for local recurrence and recurrence-free survival with these wide indications in 93 patients with primary GCTB and 30 patients with recurrent GCTB.

## Patients and methods

**Figure 1.**
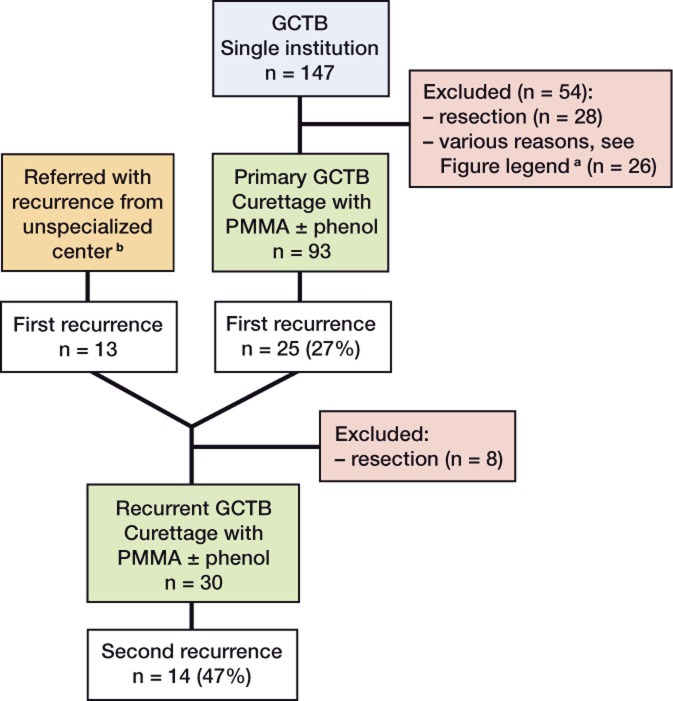
Flow chart of patients with primary and recurrent GCTB **^a^** Excluded were patients primarily treated with curettage without PMMA (n = 10), systemic treatment (n = 5), embolization (n = 1), or follow-up of less than 24 months (n = 10). **^b^** Patients who were primarily treated for GCTB by curettage with adjuvants in a center not specialized in orthopedic oncology, and who were later referred with a first recurrence to our orthopedic oncology center for repeat curettage with adjuvants.

In this retrospective single-center study, we identified 147 patients with primary GCTB who had been treated at our tertiary referral center for orthopedic oncology between 1981 and 2009 (Figure 1). All patients had a minimum follow-up of 2 years. As primary treatment, curettage with adjuvants (n = 113, 77%), en bloc resection (n = 28, 19%), or other treatment (n = 6, 4%) was performed. We did not evaluate patients who were primarily treated with resection (n = 28); nor did we evaluate patients primarily treated with curettage without PMMA (n = 10), with bisphosphonates (n = 2), with denosumab (n = 2), with radiotherapy (n = 1), or with arterial embolization (n = 1) because it was not standard treatment protocol. There was no statistically significant difference in the patient characteristics or tumor characteristics of excluded patients and of those who were included.

### Primary GCTB

We evaluated 93 patients (55 males) who underwent curettage with adjuvants for primary GCTB. Mean age was 33 (11–61) years. In all patients, PMMA was used as cavity fill-up after curettage. Additional phenol was applied on cavity borders whenever adequate protection of surrounding tissues was possible (n = 75).

### Recurrent GCTB

We also identified 38 patients who developed local recurrence after primary curettage with adjuvants either at our center (n = 25) or elsewhere (n = 13). 8 patients who underwent resection for a first recurrence were excluded. The other 30 patients underwent re-curettage with adjuvants for recurrent GCTB at our center (17 from our center and 13 referred). Mean age was 35 (18–65) years. PMMA was used in all 30 patients, and additional phenol in 21 of them.

### Data collection

Data were collected from the medical records and they included information on age, sex, tumor location, soft tissue extension, and pathological fracture (Table 1). We defined soft tissue extension as a complete breakthrough of the cortex and additional extension into adjacent soft tissue (i.e. a tumor mass). Cortex destruction was first assessed on plain radiographs in all patients. Subsequently, extension into the surrounding soft tissues was assessed on MR imaging (unless the tumor was centrally located, confined strictly to bone, and with no cortex destruction in 2 planes on conventional radiographs). Preoperative MR imaging results were available for 84 patients and preoperative CT results for 4 patients. At our center, we had had access to an MRI scanner since 1987 but 5 patients were operated before this date.

**Table 1.** Patient demographics

	Primary GCTB (n = 93)	Recurrent GCTB (n = 30)
Sex		
Male	55	9
Female	38	21
Location		
Proximal humerus	3	1
Distal radius	13	5
Proximal femur	2	2
Distal femur	52	13
Proximal tibia	16	8
Distal tibia	5	1
Fibula	2	–
Tumor characteristics		
Soft tissue extension	25	8
Pathological fracture	19	–
Curettage with adjuvants		
PMMA and phenol	75	21
PMMA alone	18	9

19 patients had a pathological fracture at presentation. Soft tissue extension in these cases was classified in the same way as in the other cases; pathological fracture in itself was not classified as soft tissue extension. Preoperative MR imaging results were available for 17 of the 19 patients with a pathological fracture, preoperative CT results were available for 1 patient, and conventional radiographs alone were available for 1 patient. 8 of the 17 patients with preoperative MR imaging results also had a pathological fracture and a soft tissue component. Only a fissure was reported in 2 patients (no soft tissue extension), none or a slight dislocation in 10 patients (3 with soft tissue extension), and a moderate to substantial dislocation of the fracture was reported in 7 patients (5 with soft tissue extension). All the data were complete.

Mean follow-up time was 8 (2–24) years. The follow-up protocol consisted of conventional radiography at 1.5, 3, and 6 months postoperatively, followed by half-yearly radiographs until 2 years postoperatively, and then radiographs taken annually over the next 10 years. MR imaging was performed at 1, 2, 5, and 10 years.

### Statistics

Recurrence-free survival (RFS) of curettage with PMMA with or without phenol was determined (Kaplan-Meier) for primary GCTB (n = 93) and recurrent GCTB (n = 30). Differences were assessed with log-rank test. Time to recurrence was defined as time from primary surgery to the date on which a recurrence was confirmed by biopsy.

Risk factors for local recurrence after primary curettage with adjuvants (n = 93) were assessed by Cox regression analysis. Age below 30, sex, tumor location (distal radius vs. other), soft tissue extension, and pathological fractures were included. Interaction terms of variables that might interact (soft tissue extension, location, pathological fractures, and young age) were assessed by successive incorporation in the regression model. There was some evidence of interaction (interaction with soft tissue extension, p < 0.05) but the numbers in this series were too small (n = 4–10) for reliable estimation of this interaction effect, and the results are not reported. Statistical analysis was performed with SPSS.

## Results

**Figure 2.**
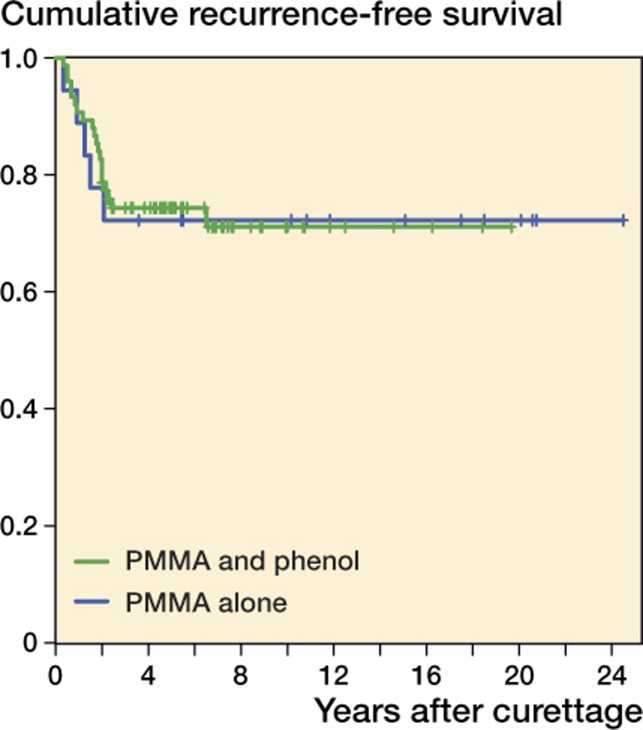
Kaplan-Meier estimated recurrence-free survival of primary GCTBs treated with curettage with PMMA and phenol (n = 75; green) or PMMA alone (n = 18; blue ) (p = 0.94).

**Figure 3.**
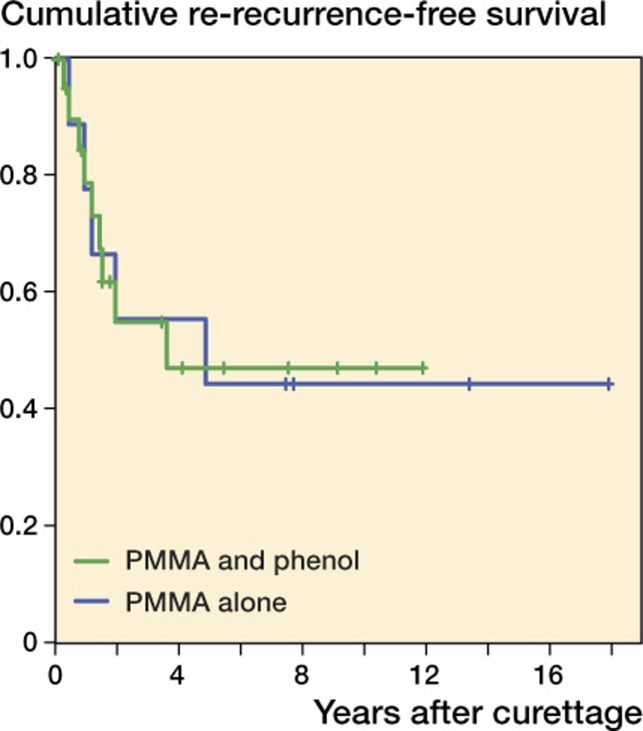
Kaplan-Meier estimated recurrence-free survival of recurrent GCTBs treated with curettage with PMMA and phenol (n = 21; green) or PMMA alone (n = 9; blue) (p = 0.99).

25 of the 93 patients with primary GCTB had a local recurrence. Mean time to first recurrence was 19 (4–78) months. Overall recurrence-free survival rates at 2 and 5 years were 0.82 and 0.74, respectively, and for recurrent GCTB they were 0.63 and 0.45 (Table 2). 4 patients died after 4–9 years, all for reasons unrelated to GCTB. None of these patients had local recurrence or metastases at final follow-up. Local control was achieved using 1 or multiple intralesional procedures in 85 of the 93 patients at 5 years postoperatively. Recurrence-free survival was similar in both primary and recurrent tumors that were treated with or without phenol in addition to PMMA (Figures 2 and 3).

**Table 2.** Recurrence-free survival for primary and recurrent GCTB

	n	Rec.	2-year RFS	95% CI	5-year RFS	95% CI	p-value
Primary							
Curettage with adjuvants	93	25	0.82	0.73–0.90	0.74	0.65–0.83	
PMMA + phenol	75	20	0.83	0.74–0.91	0.74	0.64–0.84	0.9
PMMA alone	18	5	0.78	0.59–0.97	0.72	0.51–0.93	
Recurrent							
Curettage with adjuvants	30	14	0.63	0.45–0.81	0.45	0.25–0.65	
PMMA + phenol	21	9	0.55	0.31–0.79	0.47	0.23–0.72	1.0
PMMA alone	9	5	0.56	0.23–0.88	0.44	0.12–0.77	

Rec.: local recurrence; RFS: recurrence-free survival.

Potential risk factors for local recurrence were soft tissue extension (14 of 25 recurred) and pathological fracture (5 of 19 recurred). For primary GCTB without soft tissue extension, the local recurrence rate was 12/68; the risk of local recurrence was 5 times higher in tumors with soft tissue extension (Table 3). Age below 30, sex, presence of pathological fracture, or location in the distal radius had no apparent influence on the risk of local recurrence.

**Table 3.** Potential individual risk factors for recurrence in GCTB

	n	Rec.	HR	95% CI	p-value
Potential individual risk factors					
Soft tissue extension	25	14	5	2–12	0.001
Distal radius	13	6	2	0.8–5	0.1
Pathological fracture	19	5	1.3	0.5–3.5	0.7
Age under 30	46	13	1.4	0.6–3.2	0.4
Gender	93	25	0.8	0.3–1.8	0.6
Local adjuvants **^a^**					
PMMA alone	18	5			
Phenol and PMMA	75	20	0.8	0.3–2.1	0.6

**^a^**Reference was PMMA alone in subanalysis in Cox regression analysis.

Rec.: local recurrence; HR: hazard ratio.

## Discussion

This study demonstrates that curettage with adjuvants is a feasible first-choice treatment option for GCTB, even in the case of soft tissue extension or pathologic fracture. We performed intralesional treatment in the majority of GCTBs with soft tissue extension, either alone or combined with pathological fractures. This would explain the relatively high local recurrence rate, but if we only consider GCTB without a soft tissue component, the local recurrence rate is comparable to that reported in the recent literature (Table 4). However, the risk of a second recurrence after repeat curettage was relatively high (47%).

**Table 4.** Overview of recent studies on risk factors for recurrence and surgical management of primary GCTB

	A	B	C	D	E	F	G	H	I	J
O’Donnell et al. 1994	Single	60	–	–	–	13/30	–	5/30	–	Pathological fracture, soft tissue extension
Becker et al. 2008	Multi-	256	1/48	32/65	–	15/69	13/50	–	–	Soft tissue extension **^b^**
Balke et al. 2008 **^b^**	Multi-	214	0/18	32/55	–	19/52	–	7/39	5/42	Soft tissue extension, distal radius **^a^**
Kivioja et al. 2008	Multi-	294	11/92	24/47	–	32/147	–	–	–	Age **^b^**
Klenke et al. 2011	Single	118	1/22	7/22	11/32	–	–	0/1	6/40	Age **^a^**
Current study 2012	Single	93	–	–	–	5/18	20/75	–	–	Soft tissue extension

**^a^** Risk analysis performed using the whole patient population, including resections.

**^b ^**In the study, H_2_O_2_ was used as alternative to phenol. A Center B Total C Resection **^c^** D No adjuvants **^c^** E Phenol + burr **^c^** F PMMA **^c^** G PMMA + phenol **^c^** H PMMA + burr **^c^** I PMMA + phenol + burr **^c^** J Risk factors **^c ^**Number of recurrences/total number of patients.

The survival rates were similar for curettage with PMMA and phenol and for curettage with PMMA alone. The beneficial effect of using additional phenol has been debated (Kivioja et al. 2008, Klenke et al. 2011). Phenol may have a limited additive effect on the recurrence rate; however, the risk-reducing effect of PMMA may be of greater importance.

We found a 5-times higher risk of recurrence of tumors with soft tissue extension. This confirms the previously reported increase in recurrence risk with soft tissue extension: hazard ratio 2.7 (p = 0.007) (Becker et al. 2008) and likelihood ratio 4.0 (p = 0.05) (Balke et al. 2008). This can be explained by technical difficulties in the complete removal of tumor tissue when performing intralesional treatment and the lack of adequate applicable local adjuvants, in the presence of soft tissue extension.

Previously proposed risk factors—age below 30, location in the distal radius, or pathological fracture—could not be identified as risk factors for local recurrence in the present study. This may be due to the fact that most studies analyzing risk factors for local recurrence have included both intralesional treatment and resection, with a lower expected overall recurrence rate (Balke et al. 2008, Becker et al. 2008, Kivioja et al. 2008, Klenke et al. 2011).

Young age has been suggested to be a risk factor (Kivioja et al. 2008, Klenke et al. 2011), but it may in part be explained by selection bias. Younger patients could have been selected to have intralesional treatment instead of resection. We could not confirm location in the distal radius as a risk factor for local recurrence, as has been suggested previously. Balke et al. (2008) found that 8 out of 9 GCTBs located in the distal radius recurred, as compared to 6 of 13 in our study. However, 6 of their cases had soft tissue extension and in 5 cases no PMMA was applied, which may have increased the local recurrence risk. Finally, we found no correlation between recurrence risk and pathological fracture as reported by O’Donnell et al. (1994). This indicates that curettage with adjuvants could be a feasible treatment option for GCTBs with a pathological fracture (Dreinhofer et al. 1995, Deheshi et al. 2007).

In the near future, the treatment strategy could be changed given promising results from systemically targeted neoadjuvant therapy with the RANKL inhibitor denosumab (Kostenuik et al. 2009, Thomas et al. 2010). With this therapy, calcification of affected soft tissues occurs, which may extend the indications for curettage with adjuvants.

In summary, curettage should at least include PMMA as local adjuvant, and the role of phenol after curettage with PMMA as local adjuvant is questionable. The recurrence risk after curettage with adjuvants is only increased with soft tissue extension—but not with age below 30, pathological fractures, or location in the distal radius.
